# The Impact of Non-Extraction Orthodontic Treatment on the Oral-Health-Related Quality of Life between a Modified Aligner Appliance with Ni-Ti Springs and the Traditional Fixed Appliances: A Randomized Controlled Clinical Trial

**DOI:** 10.3390/medicina60071139

**Published:** 2024-07-15

**Authors:** Ziad Mohamad Alhafi, Mohammad Y. Hajeer, Youssef Latifeh, Alaa Oudah Ali Almusawi, Ahmad S. Burhan, Tareq Azizia, Samer T. Jaber, Nada Rajeh

**Affiliations:** 1 Department of Orthodontics, Faculty of Dentistry, University of Damascus, Damascus P.O. Box 30621, Syria; ziad.alhafy@gmail.com (Z.M.A.); dr.burhan-a@hotmail.com (A.S.B.); tarek.azizieh94@gmail.com (T.A.);; 2 Department of Orthodontics, School of Dentistry, University of Jordan, P.O. Box 2212, Amman 11942, Jordan; 3 Department of Internal Medicine, Faculty of Medicine, University of Damascus, Damascus P.O. Box 30621, Syria; yoseflatifa@gmail.com; 4 Department of Orthodontics, Faculty of Dentistry, Al Knooz University, Basrah AM86 BR9, Iraq; alaaalmosawi2012@gmail.com; 5 Department of Orthodontics, Faculty of Dentistry, Al-Wataniya Private University, Hama P.O. Box 1113, Syria; samerjaber1989@hotmail.com

**Keywords:** oral-health-related quality of life, aligners, mild crowding

## Abstract

*Background and Objectives*: To compare the oral-health-related quality of life (OHRQoL) outcomes between patients treated with modified Ni-Ti spring-based alignment appliances or conventional fixed appliances using the Oral Health Impact Profile 14 (OHIP-14), as well as the levels of satisfaction with the appliance appearance, treatment progress, and outcomes. *Materials and Methods*: Thirty-six patients (11 males, 25 females) were randomly divided into two groups: either the modified aligner appliance with Ni-Ti springs group (MAA) or the traditional fixed appliances group (FA). The allocation ratio was 1:1, and the randomization process was carried out by an independent investigator not involved in this study. Mild crowding cases were included in this study. The OHRQoL of patients was evaluated using the short-form Oral Health Impact Profile (OHIP-14) at five time points: before the treatment commencement (T0); 2 weeks (T1), 1 month (T2), and 2 months (T3) after the treatment initiation; and post-treatment (T4). The visual analog scale (VAS) was used to evaluate the patient satisfaction. Blinding was performed only during the outcomes assessment. *Results*: This randomized controlled trial had no dropouts, and the demographic characteristics of the groups were comparable. The MAA group experienced significantly greater functional limitations compared with the FA group at all three evaluation time points (T1, T2, and T3), as evidenced by the statistically significant *p*-values (*p* = 0.004, *p* = 0.001, and *p* < 0.001, respectively). The psychological disability in the MAA group was significantly lower than in the FA group at both T2 (*p* = 0.005) and T3 (*p* = 0.003). The patient satisfaction with the appliance appearance was significantly higher in the MAA group than in the FA group (*p* = 0.002). *Conclusions*: The OHRQoL improved in both the modified aligner appliance with Ni-Ti springs and fixed appliance groups after the treatment. Moreover, the functional limitations during the treatment were less severe in the FA group, while the psychological disability was lower, and the patient satisfaction with the appliance appearance was higher in the MAA group.

## 1. Introduction

Traditionally, the primary objective of orthodontic treatment was to rectify malocclusion. However, contemporary patients prioritize psychological, social, and esthetic considerations over their oral health status [1,2,3]. Concurrently treating both function and esthetics can foster more stable psychosocial well-being [4]. The concept of oral-health-related quality of life (OHRQoL) encapsulates the impact of oral health on an individual’s daily functioning and overall quality of life [5]. Orthodontists must remain cognizant of oral health factors that influence the OHRQoL to ensure substantial improvements [6,7,8]. The OHRQoL domains provide reliable indicators for orthodontists to anticipate and assess patient needs and to guide treatment plan development decisions [9,10]. There has been a surge in demand for adult orthodontic treatment due to increased patient awareness of esthetics and improved access to orthodontic services [11,12]. Orthodontic treatment options for patients depend primarily on the diagnosis and subsequently on the severity of the malocclusion. Conventional fixed buccal appliances remain the primary orthodontic appliance used [13], with their metal brackets and wires giving them an unesthetic appearance, functional limitations, discomfort, and pain during treatment, which may impact the OHRQoL [14,15,16], which is a key consideration for patients seeking orthodontic treatment [17]. Schaefer et al. [18] identified disparities between orthodontic appliances in terms of cost, esthetics, and treatment techniques. The impact of various orthodontic appliance types on the OHRQoL is expected to be negative due to their placement and design. However, previous studies yielded varying results when assessing the influence of orthodontic treatment techniques on the OHRQoL [13,19,20]. Advancements in orthodontics have expanded the scope and potential of invisible techniques for adult patients. For most patients, esthetic appearance during orthodontic treatment is as crucial as other treatment-related factors, such as financial cost, pain, comfort, or treatment duration [13]. The past two decades have witnessed significant progress in modern orthodontics, as marked by the emergence of novel techniques and materials [20,21,22]. Consequently, numerous techniques and materials have emerged [23]. Lingual orthodontic techniques, ceramic braces, and clear aligners are among the most popular esthetic orthodontic techniques, but they are often financially costly [24,25].

The spring aligner appliance was introduced over 25 years ago by Barrier as a removable appliance for aligning mildly crowded incisors [26]. Subsequently, Don Inman modified it by incorporating nickel-titanium (Ni-Ti) springs to generate consistent and light forces on the incisor surfaces [27]. Recently, a clinical study evaluated a modified fixed type of this appliance for treating mild lower incisor crowding, demonstrating effectiveness in aligning crowded lower incisors [26]. A review of the literature revealed a lack of studies on this type of appliance and its impact on improving the patient quality of life [28,29]. Therefore, this study aimed to compare the influence of treatment with a modified aligner appliance with Ni-Ti springs and traditional fixed appliances on the patients’ quality of life during and after treatment. The null hypothesis stated that there were no statistically significant differences between the modified aligner appliance with Ni-Ti springs and traditional fixed appliances in terms of the oral-health-related quality of life during the treatment.

## 2. Materials and Methods

### 2.1. Study Design 

This trial was a single-center, two-arm, parallel-group, randomized, single-blinded controlled clinical trial with a 1:1 allocation ratio and followed the CONSORT statement guidelines [30]. It was registered at ClinicalTrials.gov (NCT06401382) and was approved by the Local Research Ethics Committee of Damascus University (ref no. DN-01062022-3). No changes occurred after the trial’s commencement. Funding was provided by the University of Damascus (ref no. 501100020595).

### 2.2. Participants, Eligibility Criteria, and Setting

This study comprised a total of 36 participants (25 females, 11 males). The patients were recruited from July 2021 to January 2022 at the Department of Orthodontics, Faculty of Dentistry, Damascus University. The inclusion criteria were patients (1) with mild crowding > 4 mm, (2) with a class I malocclusion, (3) aged between 18 and 25 years, (4) who had all lower teeth existing (except the third molars), (5) with no missing or extracted teeth (except the third molars), and (6) with good oral hygiene (plaque index > 1). The criteria for exclusion were patients (1) with a severe skeletal discrepancy, (2) who had undergone previous orthodontic treatment, (3) with bimaxillary protrusion, (4) who had systemic diseases, and (5) with bad oral hygiene. Written informed consent was obtained from all patients after the research methods and objectives were explained by distributing an information sheet to each patient.

### 2.3. Sample Size Calculation

The sample size was estimated using G*Power analysis software (G*Power Version 3.1.9.4, Kiel University, Kiel, Germany) based on an effect size estimate concerning the Oral Health Impact Profile-14 (OHIP-14) questionnaire, a study power of 0.80 to detect a two-point difference in the OHIP-14 total score, a standard deviation of 2.1 according to a previous study [31], and a significance level of 0.05. The intended statistical test was the independent *t*-test. The number of patients required for each group was 16 patients according to these assumptions. Two patients were added to each group in case of dropout, and thus, thirty-six patients (18 per group) were recruited for this study.

### 2.4. Interventions

After undergoing a clinical examination, patients who met the inclusion criteria were randomly assigned to one of two treatment groups.

#### 2.4.1. First Group: The Modified Aligner Appliance with NiTi Springs Group (MAA Group)

**Figure 1:**
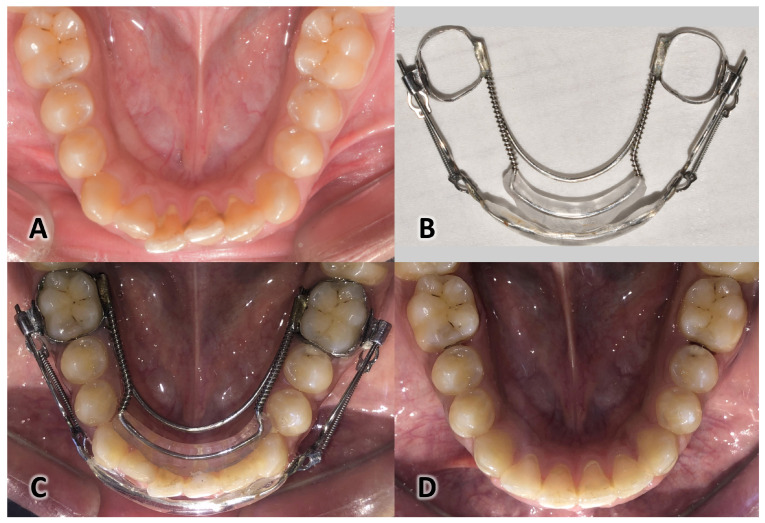
Components of the modified aligner appliance with Ni-Ti springs (MAA): (**A**) one case is shown before treatment, (**B**) the MAA components, (**C**) the MAA placed on the lower arch, and (**D**) the same case at the end of treatment after removing the MAA.

Eighteen patients were treated with the MAA, which consisted of labial and lingual pads resting on the middle part of the anterior teeth surfaces. Nickel–titanium springs in the buccal and lingual wires applied balanced and opposing forces to these surfaces when activated, resulting in the alignment of the teeth in the desired position. The appliance was made based on a predictive virtual setup which was built using the Blue Sky Plan program (version 4.7; Blue Sky Bio, Libertyville, IL, USA). The virtual digital setup was printed using a resin 3D printer (Form 3BL, Formlabs Inc., Somerville, MA, USA), and MAA was fabricated on this printed resin model; then, the appliance was applied in the lower dental arch and the springs were activated with appropriate forces (Figure 1). The patients were monitored every two weeks to observe the track of the treatment progress and adjust the springs if necessary.

#### 2.4.2. Second Group: The Fixed Buccal Appliance Group (FA Group)

Eighteen patients were treated with a traditional fixed buccal appliance. The traditional fixed appliances were chosen as the treatment for the control group, as it is considered the gold standard for the orthodontic treatment of mild crowding cases [32]. A pre-adjusted edgewise appliance (MBT prescription) with a 0.022 × 0.028-inch slot (Master Series^®^, American Orthodontics, Sheboygan, WI, USA) was used. Orthodontic archwires were utilized in the following sequence: 0.012 in NiTi; 0.014 in NiTi; 0.016 in NiTi; 0.016 × 0.022 in NiTi; 0.016 × 0.022 in stainless steel; and finally, 0.017 × 0.025 in stainless steel wire (American Orthodontics, Sheboygan, WI, USA). The patients were followed up every two weeks from the start of treatment in order to monitor the progress of leveling and alignment and make required adjustments during the treatment. The archwire was replaced when the used wire became near neutral or neutral. 

In both groups, an interproximal reduction (IPR) was performed from canine to canine before starting the treatment to secure the required space for aligning the teeth, depending on the requirements of each case, using double-sided metal abrasion strips (Interproximal Diamond Strips, Ortho Technology Inc., West Columbia, SC, USA). The treatment was considered complete once the alignment of the teeth met the guidelines of the American Board of Orthodontics Objective Grading System (ABO-OGS) [33].

### 2.5. Randomization, Allocation Concealment, and Blinding 

The study participants were randomized into the MAA group or FA group at a 1:1 allocation ratio using a simple computer randomization method. A list of the participants’ names was generated by a clinical researcher who was not involved in this study; then, they were distributed with the Minitab program (version 20; Minitab, LLC, State College, PA, USA) using computer-generated random numbers. Confidentiality was maintained by utilizing 36 consecutively numbered brown envelopes containing the groups in the same order as the randomly selected numbers. A total of 18 envelopes were prepared for the experimental group (MAA group) and 18 for the control group (FA group). The researcher responsible for the randomization also carried out this process. The envelopes were sealed and given to each participant upon their inclusion in this study. Furthermore, they were opened in sequence and only after being assigned to a participant. Patients could stop and exit the trial at any time during this study.

Blinding during the treatment and questionnaire application of either examiner or patient was not applicable because of the visibility of the applied appliances. However, blinding was only done during the data analysis, where the participants were given random numbers, and the outcomes assessor was unable to determine which group the patients belonged to. Therefore, all the results were evaluated blindly.

### 2.6. Outcomes (Primary and Secondary)

The primary outcome was the OHRQoL during treatment. The secondary outcomes included the patient satisfaction with the appearance of the orthodontic appliance, the progress of the treatment, and the final treatment result. 

A valid and reliable questionnaire called the Oral Health Impact Profile (OHIP) was used to evaluate the OHRQoL. The basic version of the OHIP questionnaire contains 49 questions (OHIP-49), and because it takes a long time to complete, the derived shorter 14-item version (OHIP-14) of the original questionnaire was used [34]. OHIP-14 includes seven domains (two questions per domain): functional limitation, physical pain, psychological discomfort, physical disability, psychological disability, social disability, and handicap. Each question was scored on a 5-point Likert scale (“never” = 0, “hardly ever” = 1, “occasionally” = 2, “fairly often” = 3, and “very often” = 4). The overall OHIP-14 scores can range from 0 to 56, and each domain score can range from 0 to 8 (0 to 4 for each item). Higher OHIP-14 scores refer to worse levels of OHRQoL [35]. The first questionnaire was given to patients before beginning the treatment (T0). Then, the questionnaires were given at different assessment times during treatment: two weeks (T1), one month (T2), and two months (T3) after beginning the treatment, and at the end of the treatment (T4). 

The visual analog scale (VAS) was used to measure the patient satisfaction at different stages of orthodontic treatment. Satisfaction with the orthodontic appliance appearance was assessed two weeks after the treatment began, satisfaction with the treatment progress was evaluated two months after the treatment started, and satisfaction with the final treatment result was measured immediately after the orthodontic appliances were removed. The VAS value ranges from 0 to 100, with 0 indicating the highest level of dissatisfaction and 100 indicating the highest level of satisfaction with the outcome achieved.

### 2.7. Statistical Analysis

All results were statistically analyzed using SPSS software version 26.0 (IBM Corp., Armonk, NY, USA). The researchers assessed the results using a 95% confidence interval and a significance level of 0.05. To verify that the data followed a normal distribution, a Shapiro–Wilk test was carried out. To ensure the homogeneity of the study sample in terms of gender and educational level, a chi-square test was used. The Friedman test was employed to detect statistically significant differences in the overall OHIP-14 scores within each group throughout the study period. Pairwise comparisons were conducted using the Wilcoxon signed ranks test. The seven domains of the OHIP-14 questionnaire, the overall OHIP-14 score in all time periods (T0, T1, T2, T3, T4), and the patient satisfaction with the orthodontic appliance appearance were compared between the MAA and FA groups using the Mann–Whitney U-test. The patient satisfaction with the treatment progress and the satisfaction with the final treatment result were compared between the two groups using an independent sample *t*-test.

### 2.8. Error of the Method

The intraclass correlation coefficient was used to assess the reliability for Little’s irregularity index (LII) measurements. The results show excellent ratings reliability, with intraclass correlations ranging from 0.912 to 0.955 in both groups for the LII measurements.

## 3. Results

### 3.1. Patient Recruitment and Follow-Up

**Figure 2:**
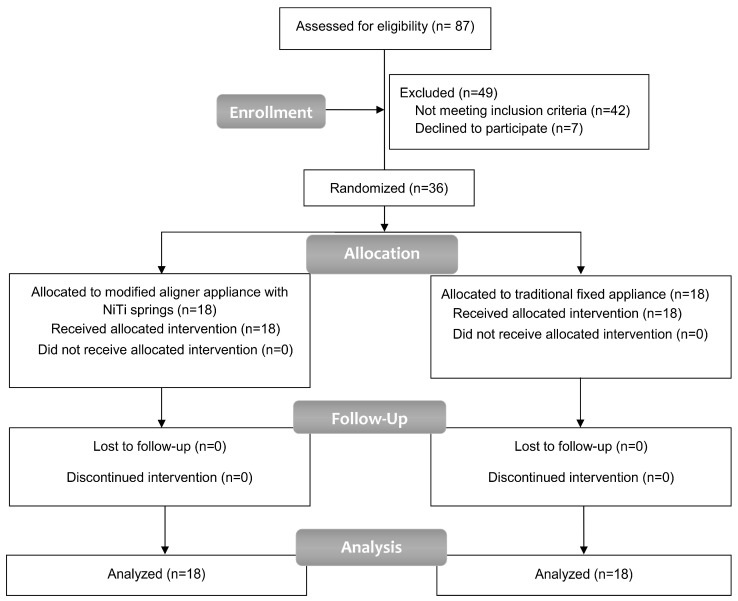
CONSORT flow diagram of patient recruitment, follow-up, and entry into data analysis.

Out of the 87 individuals assessed for eligibility, a total of 36 participants were chosen to participate in this study, and they all finished it. The attrition rate was zero. The CONSORT flow diagram of patient recruitment, follow-up, and entry into the data analysis is given in Figure 2.

### 3.2. Baseline Sample Characteristics

This study involved 36 participants who were randomly assigned into two groups: the MAA group included 13 (72.22%) women and 5 (27.78%) men with a mean age of 21.89 ± 2.63 years, whereas the FA group had 12 (66.67%) women and 6 (33.33%) men with a mean age of 20.94 ± 2.38 years. The mean of the initial LII score was 3.11 ± 0.66 and 3.01 ± 0.69 in the MMA and the FA groups, respectively. Table 1 provides a detailed overview of the participants’ sociodemographic characteristics, including age, gender, and education level.

### 3.3. Main Findings

The descriptive statistics (mean–median–quartiles) of the overall OHIP-14 score and the seven domains of the OHIP-14 questionnaire are presented in Table 2.

Initially, both the MAA and FA groups exhibited comparable means for the overall OHIP-14 scores, with values of 17.56 and 17.61, respectively. Following the placement of the appliances, the means of the overall OHIP-14 scores in both groups increased temporarily, peaking in the first two weeks of treatment: 22.72 and 19.94 for the MAA and FA groups, respectively. However, these elevated scores were short-lived, and the mean scores gradually declined over time. At the final assessment time (T4), the means of the OHIP-14 scores in both groups had fallen below their baseline (T0) levels, with values of 4.83 and 4.72 for the MAA and FA groups, respectively (*p* < 0.001, Table 3). 

There was no significant difference between the MAA group and the FA group in the overall OHIP-14 score at all assessment times (Table 4). The MAA and FA groups demonstrated comparable levels of physical pain, psychological discomfort, physical disability, social disability, and handicap at all assessment times, except for physical pain at T1, which showed a statistically significant difference (*p* = 0.001, Table 4), where the patients in the MAA group recorded higher median scores in comparison with the patients in the FA group. 

There were no significant differences between the MAA group and the FA group in the functional limitation domain at T0 (*p* = 0.830, Table 4) and T4 (*p* = 0.429, Table 4), while the functional limitation score in the MAA group was significantly higher compared with its value in the FA group at the evaluation times T1 (*p* = 0.004, Table 4), T2 (*p* = 0.001, Table 4), and T3 (*p* < 0.001, Table 4). 

For the psychological disability, there were significant differences between the two groups at T2 (*p* = 0.005) and T3 (*p* = 0.003). The scores were lower in the MAA group than those in the FA group.

The findings on patient satisfaction are detailed in Table 5. No statistically significant differences were observed between the two groups regarding the patient satisfaction with the treatment process and the treatment final result. However, significant differences were observed between the two groups regarding the patient satisfaction with the appliance appearance, which was higher in the MAA group than in the FA group (*p* = 0.002).

### 3.4. Harms

The trial proceeded without any adverse effects or unexpected consequences.

## 4. Discussion

Patient-centered care is a cornerstone of modern dentistry, recognizing that individuals have unique preferences and expectations for their oral health. OHRQoL, which is a concept that encompasses an individual’s subjective assessment of their oral health across physical, psychological, and social dimensions, should be routinely incorporated into the evaluation of patients seeking orthodontic treatment. This comprehensive approach ensures that treatment decisions are aligned with each patient’s priorities [36]. Although the modified aligner appliance with NiTi springs may influence the physical, emotional, and psychological aspects of life, there remains a paucity of published data on its effect on the oral-health-related quality of life (OHRQoL). To our knowledge, this meticulously designed randomized controlled trial was the first to comprehensively assess the impact of the MAA on the oral-health-related quality of life (OHRQoL) in mild mandibular crowding cases. This study meticulously tracked participants throughout the treatment duration by employing a standardized and validated questionnaire to accurately measure the OHRQoL changes.

The analysis of sociodemographic variables and initial LII scores revealed no statistically significant differences between the MAA and FA groups, indicating their homogeneity and comparability. The OHIP-14 questionnaire was selected as the instrument for measuring the OHRQoL due to its established prevalence in the literature [37,38]. The Arabic version of this questionnaire was validated for use among Arabic-speaking adult patients by Osman et al. [39].

### 4.1. OHRQoL Scores

The OHRQoL can be considerably improved through orthodontic treatment for patients [35,40]. Similarly, this study demonstrated a notable decrease in the overall OHIP-14 score upon treatment completion compared with the pretreatment levels, with a 72–73% reduction observed in both the MAA and FA groups. This outcome signifies a significant enhancement in the OHRQoL for all patients, regardless of the treatment modality, whether using modified aligner appliances or traditional fixed appliances. However, our results show that the overall OHRQoL levels of patients in the MAA group did not differ significantly from the FA group during the orthodontic treatment. The absence of studies that examined the MAA restricted the ability to draw comparisons with other studies. This limitation, however, could be attributed to the mild nature of the crowding cases treated in this study, which resulted in a shorter treatment duration compared with more intricate malocclusion cases.

The overall OHIP-14 scores reached their highest point two weeks after the application of orthodontic appliances. A gradual decrease was then observed during subsequent assessment times in both groups. The gradual decrease in the OHIP-14 scores for its seven items indicated improvements in the patients’ OHRQoL over time during the treatment, which can be explained by the correction of malocclusion and patients’ knowledge of the orthodontic treatment. The findings of this study align with those of Chen et al. [41] and Lai et al. [42], who found that the greatest deterioration in the OHRQoL occurred one week after the initiation of orthodontic treatment. Similarly, Gao et al. [20] reported that the OHIP-14 scores peaked on the first day after the orthodontic treatment.

The prevalence of functional limitations, including speech difficulties and impaired taste perception, was significantly higher among the patients in the MAA group compared with those in the FA group during the treatment periods (T1, T2, T3). The differences observed between the two groups immediately after the appliance was applied can be explained by the presence of the acrylic pad between the buccal surfaces of the lower incisors and the lower lip, which may have interfered with the pronunciation of some letters. The patient needed more time to adapt to its presence. When comparing this type of aligner with similar appliances in terms of placement, such as the lingual appliance, the results of this study are consistent with those of Kara-Boulad et al. [43], who found that functional limitation levels were significantly higher in the lingual appliance group compared with the traditional fixed appliance group.

During the initial two weeks of treatment, the patients in the MAA group reported significantly higher levels of physical discomfort, including pain, compared with those in the FA group. However, these differences subsided over time and were no longer statistically significant by the end of the treatment period. These differences could be attributed to the variations in biomechanical properties between the modified aligner appliance and fixed appliance. Furthermore, the lingual component of the MAA may interfere with tongue movement, which may lead to higher levels of pain and discomfort initially before the patient adapts to the appliance.

Regarding the psychological disability domain, patients in the MAA group consistently reported lower scores compared with those in the FA group. This expected outcome can be ascribed to the invisibility and enhanced esthetics of modified aligner appliances, which fostered greater treatment acceptance and improved self-esteem among the MAA group patients. This is consistent with previous studies that evaluated similar invisible orthodontic appliances, such as clear aligners [20,31,40].

### 4.2. Patient Satisfaction Levels

Compared with the FA group, patient satisfaction levels with the applied appliance appearance were statistically significantly higher in the MAA group (*p* = 0.002). This can be attributed to the fact that traditional fixed orthodontic appliances contain many visible metal components, such as brackets and wires, unlike the MAA, which includes transparent acrylic pads. This increased the patient satisfaction with the appearance of the invisible appliance during the treatment period. The results of these surveys cannot be compared with those of any other study because no previous study investigated the satisfaction of patients who have undergone treatment with this type of appliance or a similar appliance. 

The patients’ overall satisfaction with the progress and outcome of the MAA treatment was comparable with that of fixed appliances (*p* = 0.517 and 0.314, respectively). Both groups indicated that their appliances were either superior to or equal in effectiveness to their fixed appliances. This can be explained by the application of ABO-OGS guidelines, which ensures timely treatment termination for both groups [44].

## 5. Limitations

The limited scope of the current trial, which focused solely on the mandibular dental arch, hindered a comprehensive evaluation of the modified spring aligner appliance. To provide a more comprehensive assessment, future studies should investigate the appliance’s impact on the oral-health-related quality of life when applied to both the upper and lower arches.

Since this research was questionnaire-based, there is a possibility of information bias, as the results are dependent on the participants’ honesty, accuracy, and memory [45]. To minimize the risk of bias, the participants were given a comprehensive explanation of the scoring system. The current study’s sample size restricted its ability to identify small but potentially meaningful differences between the two groups. To address this limitation, future studies should involve a larger sample size, which would increase the statistical power to detect such differences. 

This study focused exclusively on mild crowding cases in adult patients who did not require tooth extraction. As a result, it could not investigate the influence of factors such as the malocclusion type, severity, age, or gender on the orthodontic treatment outcomes. To generalize the findings of this study to a broader range of orthodontic patients, future clinical trials should involve larger sample sizes and encompass a wider spectrum of malocclusion types and severities.

The absence of blinding, both for the investigators and the subjects, that arose from the intervention’s design, was a notable limitation of the trial, as it could have introduced detection bias, and thus, potentially compromised this study’s objectivity.

## 6. Conclusions

1. The initial phase of the orthodontic treatment resulted in a temporary reduction in the OHRQoL for both groups, but it peaked two weeks following the treatment commencement. Thereafter, the OHRQoL showed a gradual improvement.
2. Regardless of whether a modified aligner appliance or traditional fixed appliance was used, the patients exhibited a marked enhancement in their OHRQoL upon completion of the orthodontic treatment.
3. The MAA group had higher levels of functional limitations during the treatment than the FA group, while the FA group had higher levels of psychological disability.
4. The patients that used the MAA expressed higher levels of satisfaction with its appearance, which was approximately 50% higher than those that used the traditional fixed appliance.

## Figures and Tables

**Table 1:** Baseline sample characteristics.

	MAA Group (*n* = 18)	FA Group (*n* = 18)	*p*-Value ^†^
	Mean (SD)	Mean (SD)	
Age (years)	21.89 (2.63)	20.94 (2.38)	0.267
Initial LII	3.11 (0.66)	3.01 (0.69)	0.653
	***n*** **(%)**	***n*** **(%)**	***p*** **-Value ^‡^**
Gender			
Male	5 (27.78%)	6 (33.33%)	0.779
Female	13 (72.22%)	12 (66.67%)	
Education level			
Secondary	6 (33.33%)	8 (44.44%)	0.494
Tertiary	12 (66.67%)	10 (55.56%)	

MAA, modified aligner appliance with NiTi springs; FA, fixed appliance; LII, Little’s irregularity index; SD, standard deviation. ^†^ Employed independent sample *t*-test; ^‡^ employed chi-square test.

**Table 2:** Descriptive statistics (mean–median–quartiles) of the overall and seven domains of OHIP-14 questionnaire at all assessment time points.

OHIP-14 Domains	Group	T0		T1		T2		T3		T4	
		Mean	Md (*Q*1–*Q*3)	Mean	Md (*Q*1–*Q*3)	Mean	Md (*Q*1–*Q*3)	Mean	Md (*Q*1–*Q*3)	Mean	Md (*Q*1–*Q*3)
Functional limitation	MAA	0.39	0 (0–1)	3.78	3.5 (2.75–5)	2.33	2 (2–3)	2.17	2 (2–2)	0.83	1(1–1)
	FA	0.39	0 (0–1)	2.33	3 (1–3)	1.39	1 (1–2)	0.94	1 (0.75–1)	0.72	1 (0–1)
Physical pain	MAA	2.06	2 (0.75–3)	5.89	6 (5–7)	3.67	4 (3–4)	2.83	3 (2–3.25)	0.83	1 (0–1)
	FA	2.33	2 (1–3)	4.17	4 (3.75–5)	3.33	3 (3- 3.25)	2.72	3 (2.75–3)	0.83	1 (0–1)
Psychological discomfort	MAA	5.44	5 (5–6.25)	4.78	5 (4–6)	3.22	3 (1.75–5)	2.50	2 (2–3.25)	1	1 (0–2)
	FA	5.44	5 (4–6.25)	4.94	6 (3–6)	4.28	5 (3–5.25)	3.72	3 (3–5)	1	1 (0–2)
Physical disability	MAA	1.11	1 (0–2)	2.50	3 (1.75–3)	1.67	2 (1–2)	1.44	1.5 (1–2)	0.50	0 (0–1)
	FA	1.11	1 (0–2)	1.94	1.5 (1–2.25)	1.94	2 (1–2)	1.72	2 (1–2)	0.72	1(0–1)
Psychological disability	MAA	4.56	5 (3–6.25)	2.94	3 (2–3)	2.06	2.5 (1.5–3)	1.67	2 (0.75–2)	0.56	0 (0–1)
	FA	4.44	5 (3–6.25)	4.28	4 (3–7)	3.67	3 (3–6)	2.89	3 (2–3)	0.44	0 (0–1)
Social disability	MAA	1.72	2 (1–3)	1.50	2 (1–2)	0.72	0.5 (0–1)	0.61	0.5 (0–1)	0.39	0 (0–1)
	FA	1.50	1.5 (0.75–2.25)	1.22	1 (0–2)	0.89	1 (0–1.25)	0.56	0.5 (0–1)	0.39	0 (0–1)
Handicap	MAA	2.28	2 (0.75–4)	1.33	1 (1–2)	0.89	0.5 (0–2)	0.89	0.5 (0–2)	0.72	1 (0–1)
	FA	2.39	2 (1.5–4)	1.06	1 (0–2)	0.72	0 (0–1.25)	0.50	0 (0–1)	0.61	0.5 (0–1)
Overall	MAA	17.56	16 (13–21)	22.72	22.5 (18–25)	14.56	14 (9.75–20.25)	12.11	13 (8.75–14)	4.83	5 (3–6)
	FA	17.61	16.5 (15–20)	19.94	19.5 (15–25)	16.22	14.5 (13–20.25)	13.06	13 (11–14.25)	4.72	5 (4–5)

MAA, modified aligner appliance with NiTi springs; FA, fixed appliance; Md, median; *Q*1, first quartile; *Q*3, third quartile; T0, pre-treatment; T1, 2 weeks after initiation of treatment; T2, 1 month after initiation of treatment; T3, 2 months after initiation of treatment; and T4, post-treatment.

**Table 3:** The results of the overall OHIP-14 changes within each group.

	MAA Group				FA Group			
	*p*-Value ^†^	Pairwise Comparisons			*p*-Value ^†^	Pairwise Comparisons		
		Time	Mean Difference (95% CI)	*p*-Value ^‡^		Time	Mean Difference (95% CI)	*p*-Value ^‡^
OHIP-14. T0	*p* < 0.001 *	T0–T1	−5.16 (−7.51 to −2.81)	*p* = 0.001 *	*p* < 0.001 *	T0–T1	−2.33 (−4.74 to 0.07)	0.048
OHIP-14. T1		T1–T2	8.16 (5.41 to 10.92)	*p* = 0.001 *		T1–T2	3.72 (1.73 to 5.71)	0.002 *
OHIP-14. T2		T2–T3	2.44 (0.87 to 4.01)	0.002 *		T2–T3	3.16 (1.49 to 4.84)	*p* < 0.001 *
OHIP-14. T3		T3–T4	7.27 (5.02 to 9.53)	*p* < 0.001 *		T3–T4	8.33 (6.99 to 9.67)	*p* < 0.001 *
OHIP-14. T4		T0–T4	12.72 (9.84 to 15.61)	*p* < 0.001 *		T0–T4	12.88 (10.83 to 14.94)	*p* < 0.001 *

MAA, modified aligner appliance with NiTi springs; FA, fixed appliance; CI, confidence interval; OHIP-14, Oral Health Impact Profile-14; T0, pre-treatment; T1, 2 weeks after initiation of treatment; T2, 1 month after initiation of treatment; T3, 2 months after initiation of treatment; and T4, post-treatment. ^†^ Employed Friedman test; ^‡^ employed Wilcoxon signed ranks test * *p* < 0.01 (adjusted α = 0.05/5 = 0.01 according to Bonferroni’s correction).

**Table 4:** Comparative statistics of the overall and seven domains of the OHIP-14 questionnaire between the MAA group and the FA group.

OHIP-14 Domains	T0		T1		T2		T3		T4	
	Mean Difference (95% CI)	*p*-Value	Mean Difference (95% CI)	*p*-Value	Mean Difference (95% CI)	*p*-Value	Mean Difference (95% CI)	*p*-Value	Mean Difference (95% CI)	*p*-Value
Functional limitation	0 (−0.44 to 0.44)	0.830	1.44 (0.64 to 2.24)	0.004 *	0.94 (0.42 to 1.46)	*p* = 0.001 *	1.22 (0.76 to 1.67)	*p* < 0.001 *	0.11 (−0.17 to 0.39)	0.429
Physical pain	−0.27 (−1.5 to 0.94)	0.572	1.72 (0.83 to 2.6)	0.001 *	0.33 (−0.28 to 0.94)	0.113	0.11 (−0.35 to 0.57)	0.872	0 (−0.55 to 0.55)	0.704
Psychological discomfort	0 (−1.01 to 1.01)	0.820	−0.16 (−1.21 to 0.88)	0.496	−1.05 (−2.18 to 0.07)	0.057	−1.22 (−2.22 to −0.22)	0.011	0 (−0.63 to 0.63)	1.000
Physical disability	0 (−0.73 to 0.73)	0.894	0.55 (−0.29 to 1.4)	0.114	−0.27 (−0.94 to 0.38)	0.419	−0.27 (−0.74 to 0.18)	0.267	−0.22 (−0.71 to 0.27)	0.226
Psychological disability	0.11 (−1.4 to 1.63)	0.923	−1.33 (−2.63 to −0.03)	0.037	−1.61 (−2.64 to −0.57)	0.005 *	−1.22 (−1.99 to −0.45)	0.003 *	0.11 (−0.44 to 0.66)	0.519
Social disability	0.22 (−0.51 to 0.95)	0.534	0.27 (−0.35 to 0.91)	0.253	−0.16 (−0.83 to 0.5)	0.681	0.05 (−0.39 to 0.5)	0.874	0 (−0.47 to 0.47)	1.000
Handicap	0.11 (−1.8 to 0.96)	0.806	0.27 (−0.34 to 0.9)	0.369	0.16 (−0.83 to 0.81)	0.590	0.38 (−0.18 to 0.96)	0.238	0.11 (−0.35 to 0.57)	0.577
Overall	−0.05 (−3.22 to 3.11)	0.656	2.77 (−0.8 to 6.36)	0.127	−1.66 (−4.95 to 1.61)	0.382	−0.94 (−3.02 to 1.13)	0.329	0.11 (−1.02 to 1.25)	0.228

MAA, modified aligner appliance with NiTi springs; FA, fixed appliance group; CI, confidence interval; T0, pre-treatment; T1, 2 weeks after initiation of treatment; T2, 1 month after initiation of treatment; T3, 2 months after initiation of treatment; and T4, post-treatment. * *p* < 0.00625 (adjusted α = 0.05/8 = 0.00625 according to Bonferroni correction).

**Table 5:** Comparative statistics of the patient satisfaction between the MAA and FA groups.

	MAA Group (*n* = 18)	FA Group (*n* = 18)	MAA versus FA	
	Mean (SD)	Mean (SD)	Mean Difference (95% CI)	*p*-Value ^†^
Treatment progress satisfaction	81.11 (13.43)	77.89 (15.98)	3.22 (−6.78 to 13.22)	0.517
Final result satisfaction	87.06 (5.85)	84.67 (7.99)	2.38 (−2.35 to 7.13)	0.314
	**Median (IQR Range)**	**Median (IQR Range)**	**Mean Difference (95% CI)**	***p*-Value ^‡^**
Appliance appearance satisfaction	91 (86–100)	45 (19–90)	39 (20.8 to 57.2)	0.002 *

MAA, modified aligner appliance with NiTi springs; FA, fixed appliance group; CI, confidence interval; ^†^ employed independent sample *t*-test; ^‡^ employed Mann–Whitney U test; * *p* < 0.00625; IQR range: the range between the first and the third quartiles.

## Data Availability

The data used and analyzed during the current research are available from the corresponding author upon reasonable request.
